# Application and Research of Left Bundle Branch-Optimized Cardiac Resynchronization Therapy in Ischemic Cardiomyopathy

**DOI:** 10.31083/RCM26240

**Published:** 2025-03-21

**Authors:** Denghong Zhang, Mingjian Lang, Benjamin Samraj Prakash Earnest, Ihab Elsayed Mohamed Ali Abdou

**Affiliations:** ^1^Department of Cardiovascular Medicine (Chengdu Institute of Geriatric Diseases), The Fifth People's Hospital Affiliated to Chengdu University of Traditional Chinese Medicine, 611137 Chengdu, Sichuan, China; ^2^School of Medicine, Faculty of Health and Medical Sciences, Taylor’s University Lakeside Campus, 47500 Subang Jaya, Selangor Darul Ehsan, Malaysia

**Keywords:** ischemic cardiomyopathy, heart failure, cardiac resynchronization therapy, left bundle branch-optimized cardiac resynchronization therapy

## Abstract

**Background:**

This study aimed to evaluate the effectiveness of left bundle branch-optimized cardiac resynchronization therapy (LOT-CRT) in patients diagnosed with heart failure and reduced ejection fraction due to ischemic cardiomyopathy.

**Methods:**

A total of 78 patients with ischemic cardiomyopathy who underwent pacemaker implantation at a single center between March 2020 and March 2022 were randomly assigned to two groups based on different pacing methods: LOT-CRT group (n = 39) and biventricular pacing (BVP) group (n = 35). Pacing threshold, impedance, electrocardiogram QRS wave duration during pacing, ventricular pacing ratio during follow-up, and cardiac ultrasound-related indicators were compared immediately after surgery and at the six-month follow-up.

**Results:**

The two groups were similar regarding baseline characteristics, cardiac ultrasound and magnetic resonance imaging (MRI) parameters, and overall cardiac function. However, the BVP group demonstrated higher pacing thresholds and impedance levels immediately after surgery and at the six-month follow-up (*p* < 0.001). Moreover, the X-ray exposure time was significantly longer in the BVP group compared to the LOT-CRT group. While no significant differences in QRS duration were observed between the groups preoperatively, the QRS duration in the LOT-CRT group was significantly shorter both immediately after surgery and during follow-up (*p* < 0.001). No significant differences were found between the groups in terms of the New York Heart Association (NYHA) functional class, left ventricular ejection fraction (LVEF), or left ventricular end-diastolic diameter (LVEDD). Six months post-surgery, both groups showed modest improvements in NYHA class, LVEF, and LVEDD, with the LOT-CRT group demonstrating significant improvements (*p* < 0.001).

**Conclusions:**

LOT-CRT may be an alternative treatment for patients with heart failure complicated by left bundle branch block due to ischemic cardiomyopathy in whom BVP is ineffective.

## 1. Introduction

Cardiac resynchronization therapy (CRT), which typically involves biventricular pacing (BVP), is a crucial treatment for patients with cardiomyopathy, left bundle branch block (LBBB), and advanced heart failure (HF). However, up to 30% of patients do not respond to BVP, particularly those with ischemic cardiomyopathy (ICM). Consequently, exploring alternative treatments for these patients is essential to improve clinical outcomes. Recent studies indicate that physiological left bundle branch pacing (LBBP) can significantly reduce or even normalize the width of QRS waves and improve clinical outcomes [1, 2, 3, 4, 5]. Furthermore, other studies have shown that left bundle branch-optimized cardiac resynchronization therapy (LOT-CRT) can improve the prognosis for patients with non-ischemic cardiomyopathy (NICM) [6, 7, 8]. However, more comprehensive research is needed to examine the efficacy of LOT-CRT in patients with ICM. Therefore, this study aimed to investigate the therapeutic effect of LOT-CRT and provide a theoretical foundation and valuable insights for applying LOT-CRT in these patients.

## 2. Materials and Methods

### 2.1 Research Object 

This prospective, randomized study was conducted at the People’s Hospital affiliated with Chengdu University of Traditional Chinese Medicine from March 2020 to March 2022. Patients with ICM who met the following criteria were eligible for inclusion: age range of 18 to 65 years; conformity with CRT criteria: New York Heart Association (NYHA) functional classes III–IV, electrocardiogram showing complete LBBB, QRS interval >120 ms, and left ventricular ejection fraction (LVEF) ≤35%. All enrolled patients received at least three months of guideline-directed drug therapy [9]. The exclusion criteria were as follows: patients who did not meet the diagnostic criteria for ICM; patients requiring an upgrade from the common pacemaker to CRT; severe liver and kidney insufficiency; life expectancy <1 year [10, 11]; patients unwilling to participate in the study.

During the study period, 78 patients who met the inclusion criteria were randomly assigned to the LOT-CRT (n = 39) and BVP (n = 39) groups using a random number table. Overall, from the originally assigned 39 patients in the BVP group, two patients were reassigned to the LOT-CRT group; one abandoned surgery, and another experienced a surgical failure; thus, 35 patients were included in the BVP group. Comparatively, for the LOT-CRT group, two patients experienced surgical failure and were excluded; however, since two patients were reassigned from the BVP group, the number of patients in the LOT-CRT group remained at 39. This study was approved by the Medical Ethics Committee of the Fifth People’s Hospital, Affiliated with the Chengdu University of Traditional Chinese Medicine (Ethics Number: Ethical review 2022-009 (Section) -01). Written informed consent was obtained from all patients before their enrolment. This study was conducted in accordance with the guidelines of the Declaration of Helsinki. Data were anonymized during analysis and reporting to protect the privacy of participants.

### 2.2 Randomization Procedure

Patients were randomly assigned to either the LOT-CRT or BVP groups using a computer-generated random number table. The allocation was performed in a 1:1 ratio. To ensure anonymity, the randomization process was managed by an independent coordinator not involved in patient care or the follow-up assessment. The assignment was sealed in opaque envelopes and opened after the patient met the inclusion criteria and provided informed consent. This randomization method was implemented to minimize selection bias and ensure comparable baseline characteristics between the two groups.

### 2.3 Research Method

All patients were treated for chronic HF using angiotensin-converting enzyme inhibitors (ACEIs)/angiotensin receptor antagonists (angiotensin receptor blocker, ARB) and β receptor blockers as per the clinical guidelines. Diuretics were administered depending on the state of fluid retention. All patients received standard medication for at least three months [12, 13, 14]. All surgeons involved in the procedure had prior experience with CRT implants, having completed a minimum of 50 LBBP implants. The LBBP procedure was carried out using the SelectSecure system (Model 3830 Lead, 69 cm; C315 His sheath, Medtronic, Minneapolis, MN, USA).

### 2.4 Implantation Procedure 

The 3830 pacing lead was positioned in the right anterior oblique 30° fluoroscopic view via the C315 His sheath. Unipolar pacing was conducted at 2.0 V/0.4 ms to identify the optimal pacing site based on the following criteria: (1) The pacing QRS duration in lead V1 with the 3830 lead tip should exceed 120 ms and display a “W” morphology, with a notch observed at either the nadir or upstroke; (2) The R-wave amplitude at the tip electrode should be at least 5.0 mV. The 3830 lead was then rotated clockwise, approximately five to six turns, with unipolar pacing applied at each rotation to dynamically assess QRS morphology, QRS duration (QRSd), pacing impedance, and R-wave amplitude. As the lead approached the left bundle branch (LBB) region, a marked reduction in QRSd was observed. The left ventricular peak time was assessed in leads V5 to V6. Rotation was stopped once the left ventricular peak time significantly shortened and stabilized across different pacing outputs (>5.0 V/0.4 ms and 2.0 V/0.4 ms). Pacing and fluoroscopic assessments were performed at a 45° left anterior oblique position to gauge the depth of septal penetration. Both unipolar and bipolar pacing tests were conducted, and the LBB potential was evaluated using the intracardiac electrogram. Fluoroscopy was set to 4 frames per second, with cine imaging recorded at 7.5 frames per second.

**Fig. 1.**
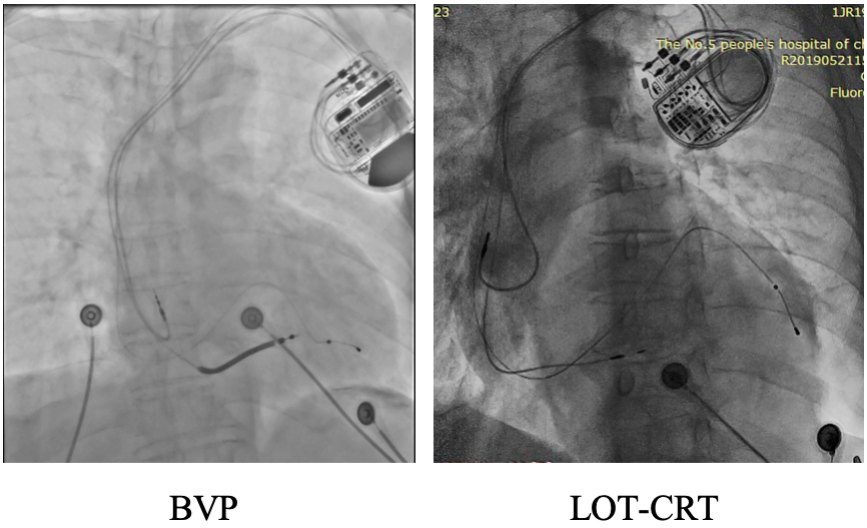
**Representative chest X-rays from the first postoperative day are shown for both groups**.

Traditional CRT was performed via the axillary vein approach. A balloon catheter was inserted, and after retrograde venography delineated the course of the coronary vein, the left ventricular lead was advanced through the coronary sinus sheath to the distal lateral or posterolateral branch of the coronary sinus. After obtaining a satisfactory threshold and sensing parameters, the pacing was performed at 10 V, with a pulse width of 1.0 ms to prevent diaphragmatic stimulation. The right ventricular apical and right atrial leads were then implanted sequentially using conventional methods. Finally, the pacemaker generator was connected to the leads and placed into a preformed subcutaneous pocket. The incision was then sutured in layers. The pocket was closed and covered with a sterile dressing, and local pressure was applied for six to eight hours using a sandbag (Fig. 1). All patients underwent transvenous implantation of a biventricular pacemaker, with no cases requiring open-chest implantation for the left ventricular lead. Postoperative CRT programming was optimized to the dual-chamber demand (DDD) pacing or dual-chamber demand rate-adaptive (DDDR) pacing mode, with an atrioventricular (AV) sensing interval of 100 ms and an AV pacing interval of 130 ms. The lower pacing rate was adjusted to achieve a BVP ratio of >90%, while the upper rate was set between 120 and 130 bpm. In cases where the LBBP successfully corrected the LBBB, the LBBP was applied independently, with a maximum V–V delay of 80 ms. The right ventricle (RV) or left ventricle (LV) lead output was set to 0.5 V for 0.1 ms to avoid RV or LV pacing. In instances where only LBBP was used intraoperatively, and the QRS duration exceeded 140 ms, sequential pacing, including LBBP and coronary sinus left ventricular (CS-LV) pacing, was employed, with programmed LV–RV (V–V) intervals to shorten the QRS duration further. For patients in sinus rhythm, the atrioventricular (A–V) delays were adjusted to optimize the electrocardiographic performance. The operating surgeon regularly modified the A–V and V–V intervals in patients receiving BVP to reduce the QRS duration. When QRS shortening was insufficient, echocardiographic optimization was utilized to refine the A–V and V–V intervals.

### 2.5 Surgical Standardization and Consistency

To ensure consistency in surgical techniques across patients, we implemented a comprehensive CRT implantation protocol in this study. All procedures were performed by experienced surgeons who had completed at least 50 LOT-CRT implantations. The surgical team held regular meetings to ensure strict adherence to the established protocol by all participating surgeons. Key steps in the procedure, including accurate positioning of the left bundle branch, control of implantation depth, and intraoperative monitoring and adjustment of QRS duration, were standardized using real-time imaging and electrophysiological evaluation during surgery. Additionally, pacing parameters during follow-up, including pacing threshold, impedance, and QRS duration, were uniformly recorded and analyzed to maintain consistency in postoperative outcomes.

### 2.6 Outcome Indicators

All patients were monitored through follow-up visits at the arrhythmia outpatient clinic every three months. Diuretics and digitalis were gradually reduced during these visits if the patient’s HF symptoms significantly improved. The dosages of β-blockers, spironolactone, ACEIs/ARBs, or angiotensin receptor-neprilysin inhibitors (ARNIs) were maintained unchanged during the first six months of follow-up. Data on R-wave amplitude, capture threshold, impedance, ventricular pacing percentage, and 12-lead electrocardiogram (ECG) were recorded at baseline and subsequent follow-up visits. Regular follow-ups were also performed to monitor for any electrode-related complications. QRSd was measured in the lead V1 at both the time of implantation and during follow-up visits. Echocardiography, conducted by an experienced sonographer, was performed at baseline and again at six months post-surgery. Left ventricular ejection fraction (LVEF) was calculated using the biplanar Simpson method from two-dimensional transthoracic echocardiography, with the sonographer blinded to all clinical data. Functional status was assessed using the NYHA classification, and plasma N-terminal pro-brain natriuretic peptide (NT-proBNP) levels were measured at each follow-up visit. The incidence of rehospitalization for HF and mortality were documented throughout the follow-up period. A positive response to CRT was defined as an improvement to NYHA grade 1 and an increase in echocardiographic LVEF of 5%.

### 2.7 Outcome Measures

The primary outcome of this study was to analyze the improvement in LVEF at six months post-procedure, chosen due to its strong association with long-term prognosis in heart failure patients.

The secondary outcomes included the following: QRS duration was measured at baseline, immediately after the procedure, and six months post-procedure. NYHA functional class: assessed at baseline and six months post-procedure. The left ventricular end-diastolic diameter (LVEDD) was measured by echocardiography at baseline and six months after the procedure. Plasma NT-proBNP levels were measured at baseline and six months as a marker of the severity of heart failure. The incidence of arrhythmic episodes was monitored throughout the follow-up period. Rehospitalization for heart failure and all-cause mortality were recorded as clinical outcomes at the follow-up.

### 2.8 Statistical Analysis

Statistical analysis was performed using SPSS 19.0 software (IBM Corp., Armonk, NY, USA). Continuous variables with normal distribution are presented as the mean ± standard deviation, and between-group differences were assessed using the independent two-sample *t*-test. For non-normally distributed continuous variables, data are expressed as the median (interquartile range) and analyzed by the Wilcoxon rank sum test. Categorical variables are reported as the frequency (percentage) and compared using the χ^2^ test. A *p*-value of <0.05 was considered statistically significant. A multivariable regression analysis was conducted to address potential confounding variables. Meanwhile, variables such as age, gender, and baseline LVEF were included to adjust for their possible impact on the outcomes. This method helps reduce the influence of residual confounding, leading to more reliable conclusions. The regression model was utilized to examine the relationship between the pacing method (LOT-CRT vs. BVP) and key outcome measures, including changes in LVEF, NYHA class, and QRS duration.

## 3. Results

### 3.1 Preoperative Baseline Characteristics

The mean age in the LOT-CRT group was 55.8 ± 10.0 years, and the mean LVEF percentage was 26.00 ± 4.32%. The mean age in the BVP group was 56.5 ± 10.4 years, and the mean LVEF percentage was 26.83 ± 4.17. There were no significant differences between the groups regarding sex, age at pacemaker implantation, NYHA cardiac function classification, LVEF, LV end-diastolic diameter (LVEDD), left ventricular diastolic size (LVDS), mitral regurgitation area (MRA), LV end-systolic volume (LVESV), LV end-diastolic volume (LVEDV), usage of ACEIs/ARBs, β-blockers, and digoxin, and LV electrode target vein selection (*p* > 0.05, Table 1). All patients received the prescribed medical treatment for at least three months.

**Table 1.** **Baseline characteristics**.

		BVP (N = 35)	LOT-CRT (N = 39)	*p*-value
Age, years		56.5 ± 10.4	55.8 ± 10.0	0.801
Male, n (%)		21 (60.0)	24 (62.0)	0.703
NYHA		2.9 ± 0.7	2.8 ± 0.9	0.955
	NYHA II, n (%)	9 (26.7)	8 (20.5)	
	NYHA III, n (%)	20 (57.1)	19 (48.7)	
	NYHA IV, n (%)	6 (17.1)	12 (30.7)	
Hypertension, n (%)		12 (34.3)	11 (28.2)	0.654
Diabetes mellitus, n (%)		6 (17.1)	8 (20.5)	0.923
Atrial fibrillation, n (%)		9 (25.7)	8 (20.5)	0.557
Baseline QRSd, ms		173.2 ± 22.3	175.5 ± 18.1	0.277
Left atrium, mm		43.3 ± 5.4	42.2 ± 5.0	0.822
LVEDD, mm		70.6 ± 8.0	71.2 ± 7.6	0.835
LVDS, mm		63.3 ± 9.0	63.7 ± 8.3	0.901
MRA, cm^2^		5.2 ± 2.4	5.2 ± 2.2	0.224
LVESV, mL		222.3 ± 95.6	222.5 ± 105.2	0.463
LVEDV, mL		300.6 ± 98.2	301.8 ± 107.6	0.367
LVEF, %		26.83 ± 4.17	26.00 ± 4.32	0.439
RV, mm		23.9 ± 5.9	24.0 ± 5.7	0.117
NT-proBNP, pg/mL		1714.5 (914.7, 2514.3)	1757.1 (997.2, 2517.0)	0.532
Drug therapy				
	Digitalis, n (%)	23 (66.7)	27 (69.0)	0.570
	Diuretics, n (%)	35 (100.0)	10 (100.0)	1.000
	ACEI/ARB, n (%)	35 (100.0)	10 (100.0)	1.000
	Mineralocorticoid receptor antagonist, n (%)	35 (100.0)	10 (100.0)	1.000
	Beta-blocker, n (%)	32 (91.4)	31 (79.0)	0.087

Note: ACEI, angiotensin-converting enzyme inhibitor; ARB, angiotensin II receptor blocker; BVP, biventricular pacing; LOT-CRT, left bundle branch-optimized cardiac resynchronization therapy; LVEDD, left ventricular end-diastolic diameter; LVEDV, left ventricle end-diastolic volume; LVEF, left ventricular ejection fraction; LVESV, left ventricular end-systolic volume; NYHA, New York Heart Association; RV, right ventricle; QRSd, QRS duration; LVDS, left ventricular diastolic size; MRA, mitral regurgitation area; NT-proBNP, N-terminal pro-brain natriuretic peptide.

### 3.2 Comparison of ECG, Pacing Characteristics, and Surgical Parameters between the Two Groups at Six Months after Surgery

At the time of implantation, the mean QRSd in the LOT-CRT group was significantly shorter than in the BVP group (*p* < 0.001). Six months post-implantation, the QRSd in the LOT-CRT group remained notably shorter than in the BVP group (114.0 ± 13.0 vs. 151.0 ± 19.2 ms, *p* < 0.001). Furthermore, significant differences were observed in the LBBP thresholds and pacing impedance between the two groups (*p* < 0.001, Table 2).

**Table 2.** **ECG, pacing characteristics, and surgical parameters at six months after surgery**.

		BVP group	LOT-CRT group	*p*-value
Variables		N = 35	N = 39	
	CRT-D, n (%)	26 (74.3)	31 (79.5)	0.865
At implantation				
	Threshold, at 0.4 ms, V	1.28 ± 0.59	0.83 ± 0.40	0.002^**^
	Paced QRSd, ms	157.6 ± 21.8	128.0 ± 16.7	<0.001^**^
	X-ray exposure duration (total), min	40.4 ± 8.7	32.6 ± 9.5	<0.001^**^
	Impedance, Ω	772.8 ± 245.4	608.2 ± 225.3	<0.001^**^
Follow-up				
	VP (%)	96.1 ± 2.2	98.3 ± 1.5	0.265
	Paced QRSd, ms	151.0 ± 19.2	114.0 ± 13.0	<0.001^**^
	Threshold, at 0.4 ms, V	1.32 ± 0.67	0.74 ± 0.30	<0.001^**^
	Impedance, Ω	726.3 ± 151.3	562.8 ± 185.4	<0.001^**^

Note: **p < 0.001. ECG, electrocardiogram; QRSd, QRS duration; CRT-D, cardiac resynchronization therapy with a defibrillator; VP, ventricular pacing.

### 3.3 Echocardiography and Clinical Findings in both Groups at Six Months after Surgery

The LOT-CRT group showed significantly greater LVEF (*p* < 0.001), a higher rate of CRT over-response (*p* < 0.001), significant improvement in NYHA cardiac grade (*p* < 0.001), a substantial reduction in plasma NT-proBNP level (*p* < 0.001), and higher CRT response rate (89.7% vs. 74.2%, *p* = 0.021). No events of HF rehospitalization or all-cause death were observed in either group at the six-month follow-up. Moreover, the differences in outcome measures between the LOT-CRT and BVP groups remained significant even after adjusting for potential confounders (age, gender, and baseline LVEF) using multivariable regression analysis. Specifically, the LOT-CRT group demonstrated a greater improvement in LVEF (adjusted *p* < 0.001), a more significant reduction in QRS duration (adjusted *p* < 0.001), and better NYHA class improvement (adjusted *p* < 0.001) compared to the BVP group. These findings suggest that the observed benefits of LOT-CRT are independent of the baseline characteristics (Table 3).

**Table 3.** **Echocardiography and clinical findings at six months after surgery**.

		BVP group	LOT-CRT group	*p*-value
Variables		N = 35	N = 39	
Echocardiography parameters				
	LVEDD, mm	62.6 ± 7.5	47.4 ± 7.9	<0.001^**^
	LVEF, %	34.0 ± 5.6	55.5 ± 6.2	<0.001^**^
	Echocardiographic response, n (%)	20 (57.1)	31 (79.5)	0.033^*^
	Upper‐response, n (%)	6 (17.1)	16 (41.0)	0.001^**^
NYHA class		2.4 ± 0.6	1.2 ± 0.9	<0.001^**^
	NYHA I, n (%)	6 (17.1)	19 (48.8)	
	NYHA II, n (%)	18 (51.5)	16 (41.0)	
	NYHA III, n (%)	9 (25.7)	4 (10.2)	
	NYHA IV, n (%)	3 (8.6)	0 (0.0)	
NT‐proBNP, pg/mL		1224.3 (568.5, 2310.7)	432.9 (210.9, 709.2)	<0.001^**^
Clinical response, n (%)		26 (74.2)	35 (89.7)	0.021^*^

Note: *p < 0.05, **p < 0.001.

## 4. Discussion

Ischemic cardiomyopathy refers to the left ventricular systolic dysfunction caused by coronary artery disease (CAD), which is the most common cause of HF worldwide [15]. The five-year mortality of ICM patients with HF ranges from 50% to 84% [16]. Thus, developing individualized treatment strategies for such patients represents a key challenge in clinical practice.

BVP is an established treatment for patients with LV systolic dysfunction (LVEF <35%) and heart failure associated with LBBB-related electrical abnormalities. BVP is also the standard HF treatment recommended by current guidelines [17]. Study has shown that BVP can improve HF symptoms and ventricular function by simultaneously stimulating both ventricles [18]. However, at least 30% of patients treated with BVP may not show any therapeutic benefit, and some patients may even show a deterioration in health status related to the extent and distribution of LV scars, suboptimal site of LV electrode stimulation, gender, and electrical or mechanical desynchrony [19]. Although BVP can significantly improve hemodynamics, it has not been proven to improve the long-term prognosis of patients [20]. However, compared with BVP, LBBP has shown a considerably higher LVEF improvement [21, 22, 23] and echocardiographic super-remission rate [24, 25]. In other studies, LOT-CRT was found to significantly shorten the QRSd width and restore mechanoelectric synchronization compared with BVP, ultimately improving the clinical outcome of NICM [26, 27, 28]. In a recent study by Compagnucci *et al*. [29], the authors explored differentiating sensor changes in a composite heart failure implantable cardioverter defibrillator (ICD) monitoring index. This work highlights the variability in patient responses to CRT and underscores the need for personalized treatment strategies. Moreover, the study suggests that sensor-based monitoring can provide crucial insights into patient-specific heart failure dynamics, potentially guiding more tailored therapy adjustments. The above studies indicate the advantages of LOT-CRT over BVP in NICM patients. However, further research is needed to determine whether this pacing mode can be used routinely and to evaluate its efficacy in patients with ICM.

**Fig. 2.**
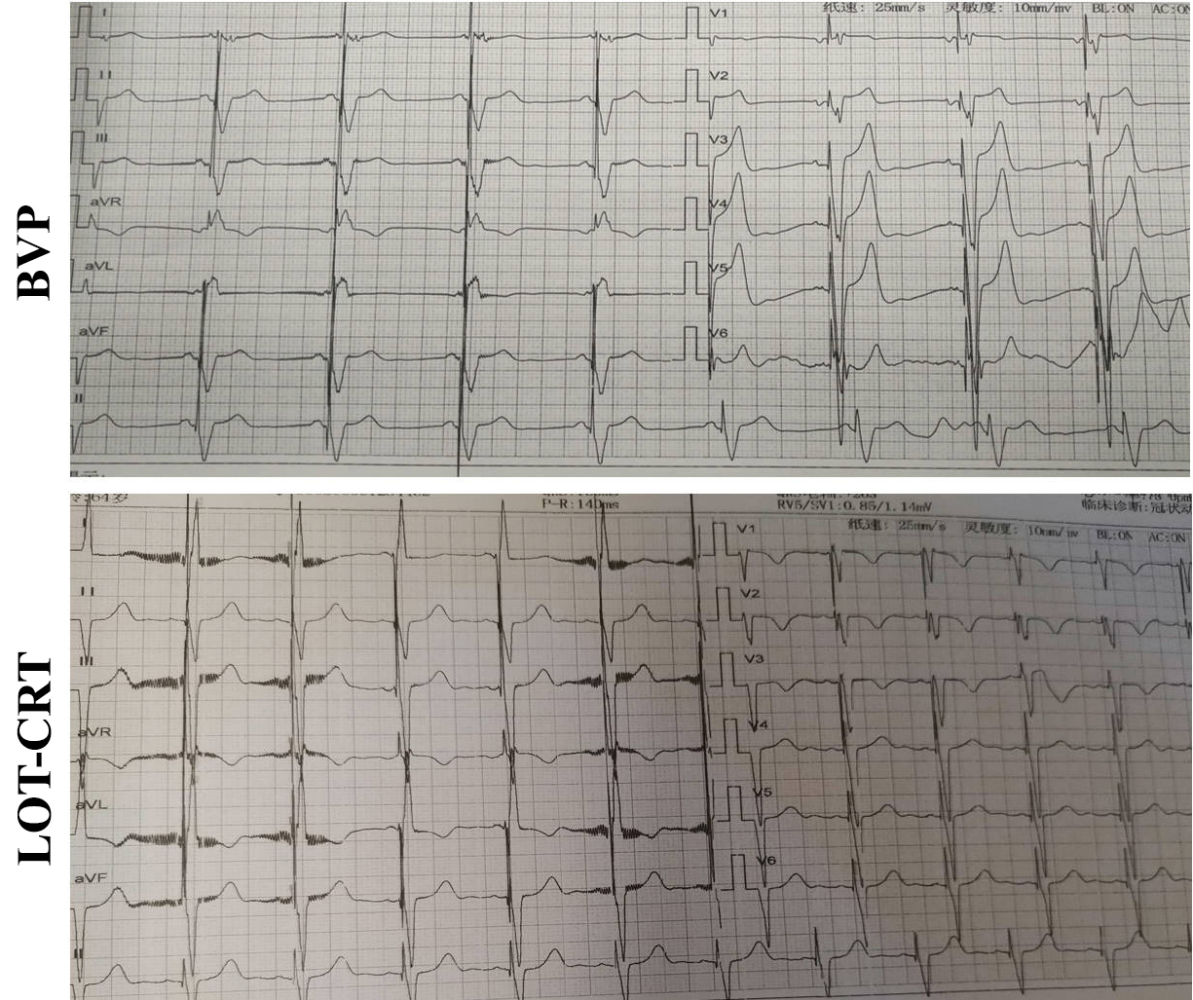
**Typical image of an ECG in the two groups at six months post-surgery**.

**Fig. 3.**
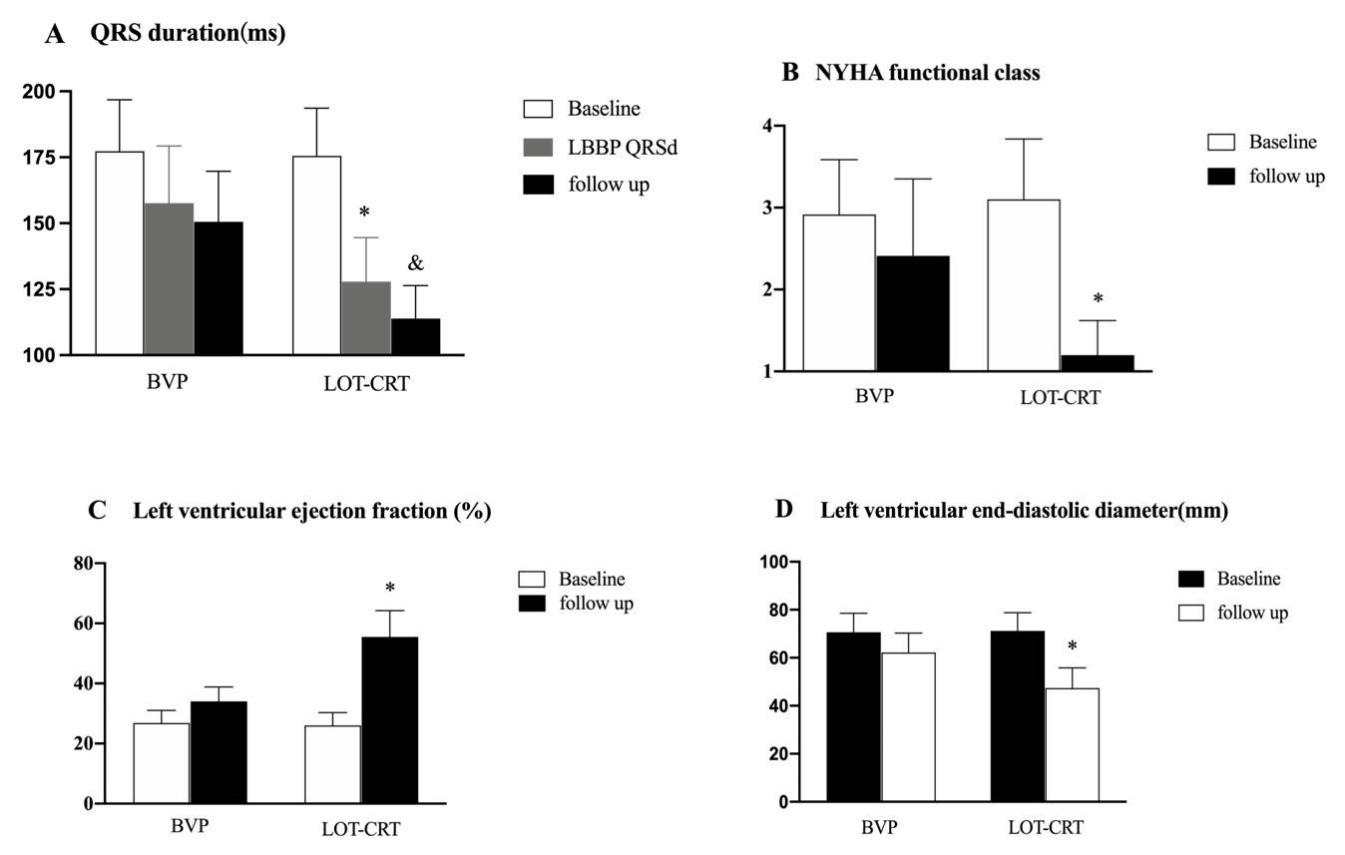
**Comparison of the QRS duration and cardiac function at baseline and six months follow-up**. (A) QRS duration at implantation and six-month follow-up were significantly shorter in the LOT-CRT group. (B,C) The NYHA cardiac function grade and LVEF at the six-month follow-up were significantly improved in the LOT-CRT group. (D) The left ventricular end-diastolic diameter at the six-month follow-up was significantly lower in the LOT-CRT group. ^&^*p* < 0.05 vs. BVP group. ^*^p < 0.05 vs. BVP group. LBBP, left bundle branch pacing.

Hence, we conducted a preliminary investigation into the application of LOT-CRT in patients with ICM-induced HF and compared it with the traditional BVP. An intention-to-treat analysis at a six-month follow-up revealed several significant findings: the LOT-CRT group exhibited lower immediate and follow-up threshold and impedance (*p* < 0.001), shorter X-ray exposure time (*p* < 0.001), and narrower QRSd (*p* < 0.001), (Figs. 2,3). Additionally, the NYHA cardiac function classification, LVEF, and LVEDD improvements were significantly better in the LOT-CRT group. This group experienced higher CRT rates and significantly lower plasma NT-proBNP levels. Our findings align with those reported by Shunmuga Sundaram Ponnusamy *et al*. [30, 31] in patients with NICM. These findings suggest that LOT-CRT can shorten the QRS time and improve cardiac function in patients with ICM who develop HF with LBBB. Furthermore, the pacing mode used in LOT-CRT is more physiological compared to BVP, making it a potential alternative for patients who do not respond effectively to BVP. By incorporating multivariable regression analysis to adjust for possible confounding factors such as age, gender, and baseline LVEF, we were able to confirm that the observed advantages of LOT-CRT over BVP in improving LVEF, reducing QRS duration, and enhancing clinical outcomes were independent of these baseline characteristics. Thus, this analytical approach strengthens the robustness of our findings and decreases the influence of residual confounding, providing more reliable evidence for the clinical utility of LOT-CRT in ischemic cardiomyopathy patients.

Furthermore, the results of our study not only demonstrate superior echocardiographic outcomes with LOT-CRT compared to BVP but also suggest the potential for a reduction in arrhythmic episodes. Indeed, it is well-known that improved mechanical synchrony, as indicated by better echocardiographic response, can reduce the burden of arrhythmias in patients with heart failure. Recent studies, such as the one by Compagnucci P, *et al*. [32], have shown that LOT-CRT is associated with a lower incidence of arrhythmic events, likely due to increased physiological pacing and improved ventricular function. These findings are consistent with our observation that LOT-CRT achieves superior LVEF improvement and greater QRS narrowing, both of which are critical in minimizing arrhythmogenic substrates. Therefore, LOT-CRT may offer a dual benefit of both improving cardiac function and reducing arrhythmia risk, particularly in patients with ischemic cardiomyopathy.

Some limitations of this study warrant consideration. This study was conducted at a single center with a small sample size and a short follow-up period, which may have introduced potential bias. Therefore, more robust, multi-center prospective studies are needed to investigate further the efficacy of LOT-CRT in HF patients with LBBB.

## 5. Limitations

While the results of this study are promising, they should be interpreted in the context of certain limitations. Notably, the follow-up period of six months, while sufficient to observe initial clinical improvements and device integration, may not fully capture long-term outcomes such as survival rates, chronic device complications, or late-stage device optimizations. Subsequently, this relatively short follow-up period limits our ability to generalize the findings to longer-term clinical scenarios where factors such as lead integrity, device longevity, and patient adaptation to the device play a more pronounced role. Therefore, future studies with extended follow-up durations are needed to validate these initial findings and provide a more comprehensive assessment of the long-term benefits and risks associated with LOT-CRT.

## 6. Conclusions

In this study, LOT-CRT in patients with ICM-induced HF with LBBB demonstrated superior echocardiographic response and clinical outcome compared to BVP. Our findings indicated that LOT-CRT may be a potential alternative to BVP in these patients. Additional research is essential to establish more definitive evidence.

## Availability of Data and Materials

The data and materials supporting the findings of this study are available from the corresponding author upon reasonable request.
